# Supplementary material for: Benchmarking genome-wide association study causal gene prioritization for drug discovery
Source: NPJ Drug Discov. 2026 Nov 14;3:56. doi: 10.1038/s44386-026-00091-0 (PMC13769767; doi:10.1038/s44386-026-00091-0)
Supplement: Supplementary file 1 [file 44386_2026_91_MOESM1_ESM.pdf]

Supplementary Figures

Figure S1: Enrichment of gene prioritization strategies for successful drug targets among nearest genes

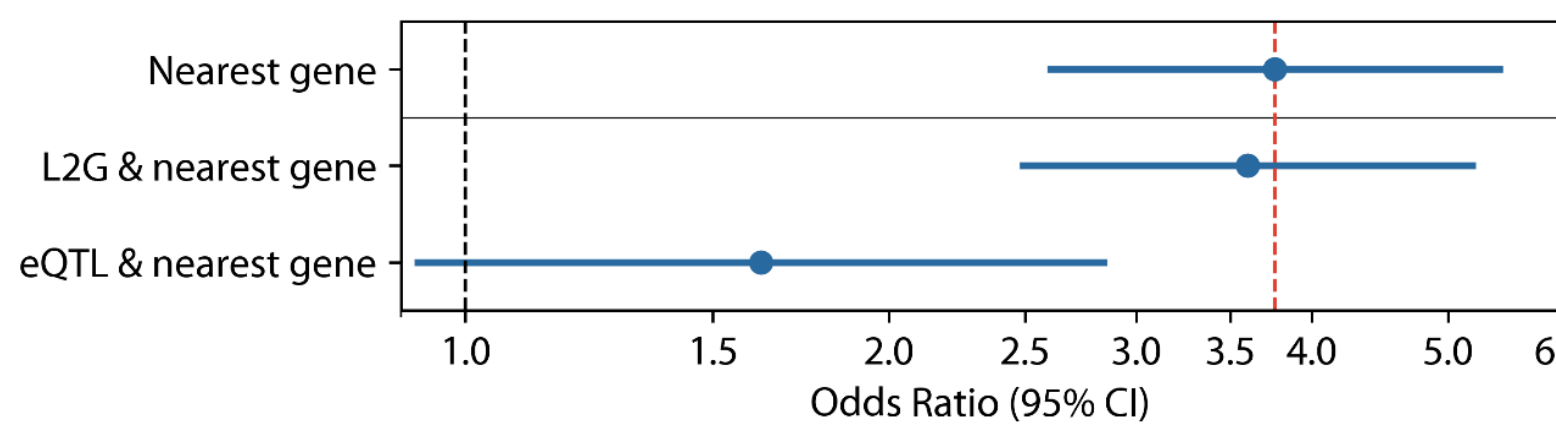

	# approved	# prioritized	OR (95% CI)	p-value
Nearest gene	56	269	3.76 (2.59, 5.47)	3.4×10^{-12}
L2G & nearest gene	51	231	3.60 (2.48, 5.23)	1.8×10^{-11}
eQTL & nearest gene	13	77	1.62 (0.92, 2.86)	0.095

Enrichment of genes prioritized by L2G and eQTL, restricted to those also identified by the nearest-gene method, among launched target-indication pairs. The dotted red line indicates the adjusted odds ratio across all nearest genes, which serves as a baseline. See Fig. 2 caption for definitions and statistical details.

Figure S2: Enrichment of gene prioritization strategies for successful drug targets stratified by whether the lead variant is coding or non-coding

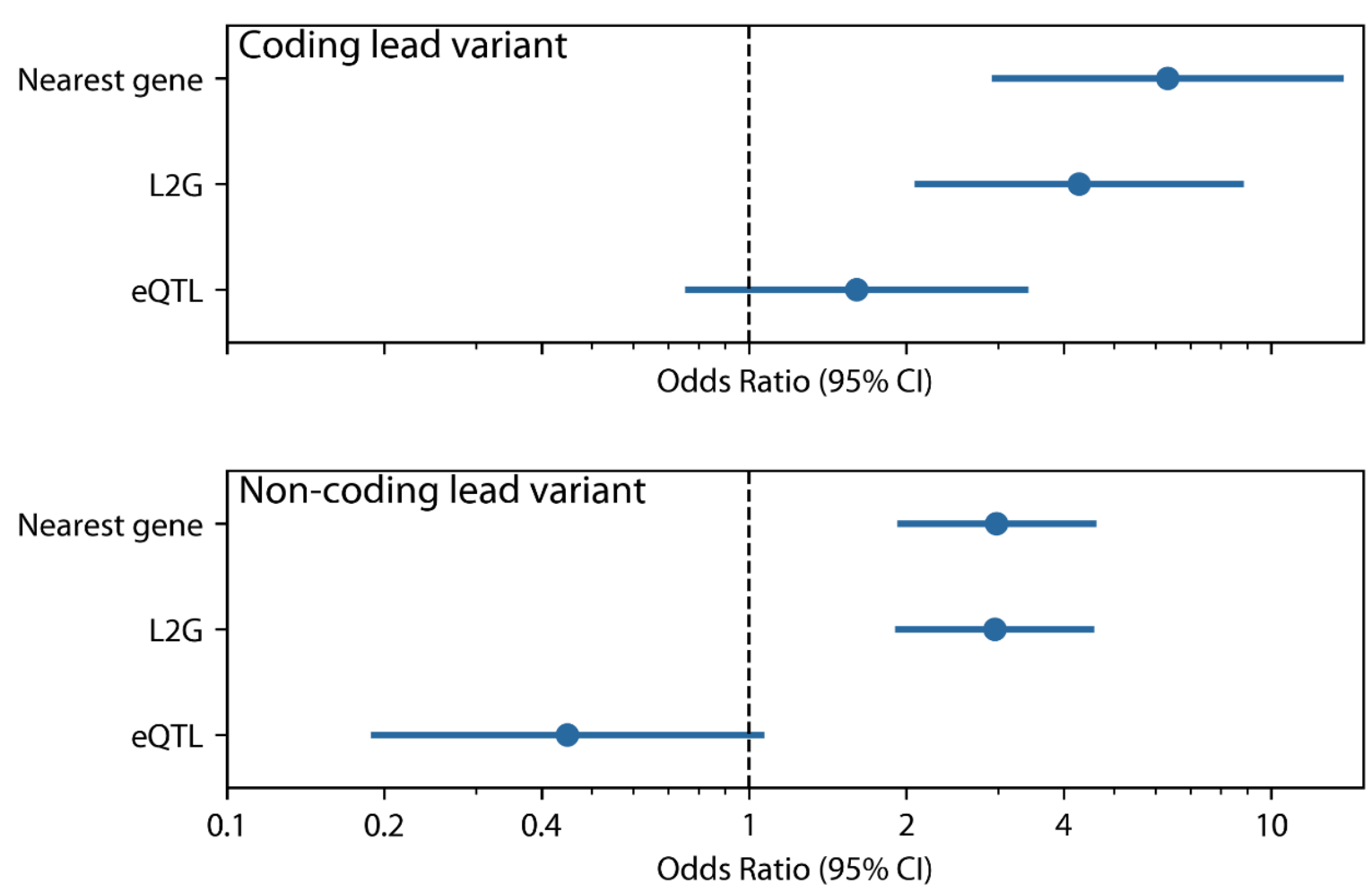

Coding lead variant	# approved	# prioritized	OR (95% CI)	p-value
Nearest gene	19	65	6.32 (2.91 - 13.73)	3.1×10^{-6}
L2G	18	62	4.28 (2.07 - 8.85)	8.9×10^{-5}
eQTL	9	40	1.60 (0.75 - 3.43)	0.22

Non-coding lead variant	# approved	# prioritized	OR (95% CI)	p-value
Nearest gene	37	204	2.98 (1.92 - 4.62)	1.1×10^{-6}
L2G	35	186	2.95 (1.90 - 4.58)	1.4×10^{-6}
eQTL	5	71	0.45 (0.19 - 1.07)	0.07

Enrichment of genes prioritized by nearest gene, L2G and eQTL colocalization among launched target-indication pairs, stratified by whether the lead variant at the associated locus is coding or non-coding. See Fig. 2 caption for definitions and statistical details.

Figure S3: Tissue-based eQTL analysis

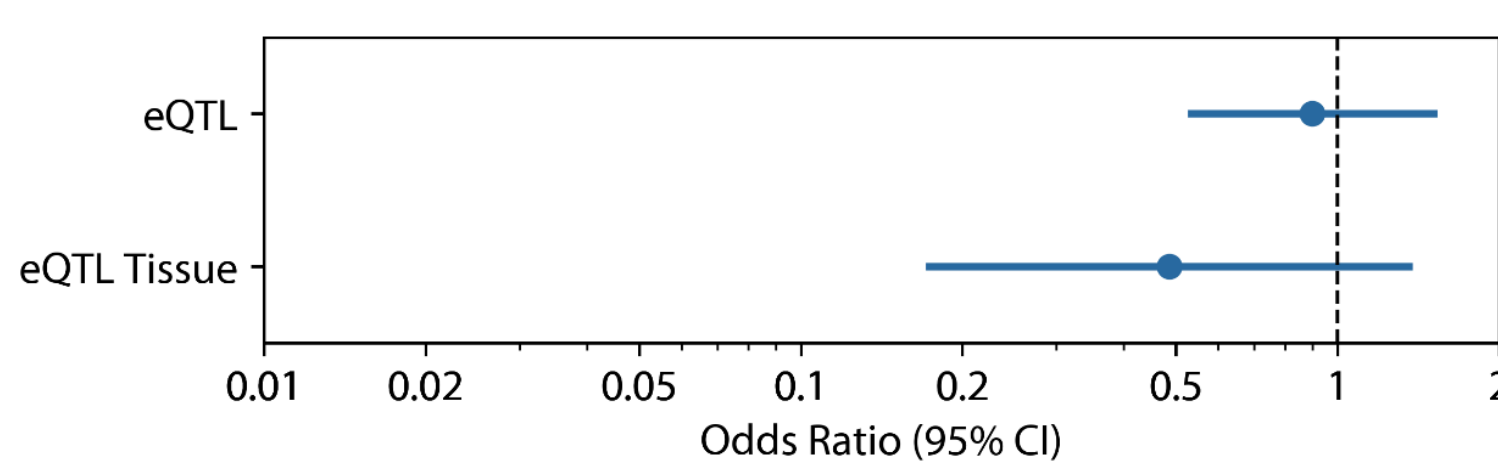

	# approved	# prioritized	OR (95% CI)	p-value
eQTL	14	111	0.90 (0.53 - 1.53)	0.69
eQTL Tissue	3	35	0.49 (0.17 - 1.38)	0.18

Enrichment of genes prioritized by tissue-based eQTL colocalization, compared to tissue-naive eQTL colocalization (i.e. the primary analysis). The tissue-based analysis restricts eQTL predictions to those where the eQTL study and GWAS study share the same high-level tissue/organ locality. See Fig. 2 caption for additional definitions and statistical details, and the Methods for the list of the 12 high-level tissue/organ localities.

Supplementary Data

Data S1: Target indication summary

The 992 target-indication pairs and their maximum clinical phase attained. MeSH term mappings to specialties included.

Data S2: GWAS selection details

The 445 GWAS retained after filtering for the best-powered disease GWAS with Medical Subject Headings (MeSH) IDs from Pharmaprojects. Credible sets and sample size are shown as indicators of study power, which were used for ranking and filtering. The source GWAS name and corresponding MeSH ID are included to document the disease-mapping scheme.
